# The association between cardiovascular health and health-related quality of life and health status measures among U.S. adults: a cross-sectional study of the National Health and Nutrition Examination Surveys, 2001–2010

**DOI:** 10.1186/s12955-015-0352-z

**Published:** 2015-09-22

**Authors:** Norrina B. Allen, Sylvia Badon, Kurt J. Greenlund, Mark Huffman, Yuling Hong, Donald M. Lloyd-Jones

**Affiliations:** Department of Preventive Medicine, Feinberg School of Medicine, Northwestern University, 680 North Lake Shore Dr., Suite 1400, Chicago, IL 60611 USA; Division of Population Health, Centers for Disease Control and Prevention, Atlanta, GA USA; Division for Heart Disease and Stroke Prevention, Centers for Disease Control and Prevention, Atlanta, GA USA

**Keywords:** Quality of Life, Cardiovascular diseases, Risk factors

## Abstract

**Background:**

This study was conducted to examine the association between ideal cardiovascular health (CVH) and health-related quality of life and health status indicators.

**Methods:**

This cross-sectional study included adult NHANES participants from 2001 to 2010 without CVD (*N* = 7115). CVH was defined according to AHA definitions with poor, intermediate and ideal levels of the seven factors (diet, BMI, physical activity, smoking, blood pressure, glucose, and cholesterol) assigned scores of 0, 1, and 2, respectively. A CVH score (CVHS) was calculated as the sum of the scores from each individual health factor (range 0–14; higher score indicating greater CVH). CVHS was categorized as poor (0–7), intermediate (8–10), and ideal (11–14). Linear regression models examined the association between CVHS category with health status and number of unhealthy days per month, adjusted for socio-demographic characteristics and disability.

**Results:**

Among US adults 20–79 years, 14, 46 and 40 % had ideal, intermediate and poor CVHS, respectively. Compared to those with poor CVH, individuals in intermediate and ideal CVH were 44 and 71 % less likely to report being in fair/poor health. Participants with ideal CVH scores reported a mean of 2.4 fewer unhealthy days over the past month, including one less day in which their physical health was not good and two fewer days in which their mental health was not good.

**Conclusions:**

Ideal CVH is associated with greater overall health status and fewer physically and mentally unhealthy days.

**Electronic supplementary material:**

The online version of this article (doi:10.1186/s12955-015-0352-z) contains supplementary material, which is available to authorized users.

## Background

Cardiovascular (CVD) disease remains the leading cause of death in the United States, accounting for nearly 800,000 deaths each year [1]. Up to 90 % of these deaths may be attributable to known and modifiable cardiovascular risk factors [2–4]. To quantify and ultimately reduce the overall burden of cardiovascular risk factors the American Heart Association (AHA) recently defined cardiovascular health [5] with seven health factors and behaviors including blood pressure, cholesterol, glucose, body mass index (BMI), smoking status, diet, and physical activity. Ideal levels of these cardiovascular health metrics are associated with reduced cardiovascular and cancer morbidity and mortality, lower healthcare costs, improved cognitive function and greater longevity [6–11].

Prior literature has demonstrated an association of individual cardiovascular risk factors with health-related quality of life [12–15]. To date, however, data are sparse on the association of CV health (CVH) as a measure of overall risk factor burden with general health status and health-related quality of life [16], which have been highlighted by the AHA as important secondary outcomes in defining CV health [5] and is also an important indicator measured by Healthy People 2020 [17]. The association between ideal cardiovascular health, objectively measured at physical exam, and HRQoL remains unknown, and it is unclear whether there are differences in this association by gender or race/ethnicity. The goal of this study was to examine the association between cardiovascular health and self-reported health status and HRQoL measures using data from the National Health and Nutrition Examination Survey (NHANES) from 2001 through 2010.

## Methods

### Study population

We used cross-sectional data from participants in NHANES, a nationally-representative survey conducted in two year cycles, from 2001 thru 2010. Participants were interviewed in their home and then invited to undergo physiologic and anthropometric examinations at a mobile examination center (MEC). NHANES participants are sampled through a complex, multi-stage sampling methodology to ensure that the sample is nationally representative. The 2001 through 2010 continuous NHANES surveys were approved by the National Center for Health Statistics Ethics Review Board, and all participants provided written informed consent. Participants aged 20 to 79 years were included in this study in order to align with the AHA 2020 strategic goals. Among 11,187 participants aged 20–79 with a fasting glucose measurement, 543 were excluded for being pregnant or breastfeeding and 969 were excluded with missing information on any component of the cardiovascular health score. We additionally excluded participants with CVD (presence of angina or ever told had heart failure, angina, coronary heart disease, heart attack, stroke; *n* = 1095), missing data for socio-demographic variables or HRQol measures (*n* = 1033) or had nonpositive sampling weights (*n* = 432). The final analytic sample was 7115 participants.

### Cardiovascular health score

The cardiovascular health score (CVHS) includes 3 health factors (total cholesterol, fasting blood glucose, and blood pressure (BP)) and four health behaviors (BMI, diet, physical activity, and smoking status). In brief, smoking status, diet and physical activity were based on participant self-report. Individuals were asked about their use of cigarettes, pipes, and cigars currently and in the past. Physical activity was assessed based on responses regarding frequency and duration of moderate- and vigorous-intensity activity. Two interviewer-administered dietary recalls were collected. Using data from the MyPyramid Equivalents Database and the methodology established by the US Department of Agriculture Center for Nutrition Policy and Promotion, each participant was assessed as to the number of dietary components they met. These five components include: consuming (1) ≥4.5 cups per day of fruits and vegetables, (2) ≥ two 3.5-oz servings of fish per week, (3) ≥ three 1-oz-equivalent servings per day of fiber-rich whole grains, (4) <1500 mg per day of sodium, and (5) ≤450 kcal (36 oz) per week of sugar-sweetened beverages. During the examination, up to three resting and seated BP measurements were made. We used the average of these BP measurements in these analyses. NHANES participants were weighed and their height measured according to a standardized protocol, from which BMI was calculated as kg/m^2^. Fasting blood samples were obtained for the measurement of total cholesterol and glucose.

Ideal, intermediate, and poor levels of each risk factor are defined in Table 1 based on the AHA 2020 Strategic Impact Goals [5]. Poor, intermediate, and ideal levels for each component were assigned a score of 0, 1, or 2, respectively. An overall CVHS was calculated as the sum of each individual component score. The CVHS thus ranges from 0 to 14, with 14 corresponding to the best CV health (and lowest burden of cardiovascular risk factors). As in previous publications, participants were categorized into poor (0–7), intermediate (8–10), or ideal (11–14) levels of CVHS [18].

**Table 1:** Ideal, intermediate and poor categories of cardiovascular health score components

Component	Score	Definition
Physical Activity		
	0	No exercise
	1	1–149 min of moderate exercise or 1–74 min of vigorous exercise/week
	2	150+ minutes of moderate exercise or 75+ minutes of vigorous exercise/week
Diet^a^		
	0	0–1 components of healthy diet
	1	2–3 components of healthy diet
	2	4–5 components of healthy diet
Glucose		
	0	≥126 mg/dL fasting
	1	100–125 mg/dL fasting or treated to <100 mg/dL
	2	<100 mg/dL fasting, unmedicated
Blood Pressure		
	0	Systolic blood pressure ≥140 mmHg or diastolic blood pressure ≥90 mmHg
	1	Systolic blood pressure 120–139 mmHg or diastolic blood pressure 80–89 mmHg or treated to <120/80 mmHg
	2	<120/80 mmHg, unmedicated
BMI		
	0	≥30 kg/m^2^
	1	25.0–29.99 kg/m^2^
	2	<25.0 kg/m^2^
Cholesterol		
	0	≥240 mg/dL
	1	200–239 mg/dL or treated to <200 mg/dL
	2	<200 mg/dL, unmedicated
Smoking		
	0	Current smoker
	1	Former smoker, quit ≤12 months ago
	2	Never smoker or quit >12 months ago

^a^Dietary components include: consuming (1) ≥4.5 cups per day of fruits and vegetables, (2) ≥ two 3.5-oz servings of fish per week, (3) ≥ three 1-oz-equivalent servings per day of fiber-rich whole grains, (4) <1500 mg per day of sodium, and (5) ≤450 kcal (36 oz) per week of sugar-sweetened beverages

### Health status and health-related quality of life

General health status based on participants’ perceived quality of health was dichotomized into fair/poor or excellent/very good/good. Health-related quality of life was determined using the validated HRQoL-4 tool developed by the Centers for Disease Control and Prevention [19]. This generic HRQoL scale for use in general health surveys compares well against the Medical Outcomes short study form (SF-36) and disease specific scales [19]. It consists of four questions about self-rated health: overall perceived quality of health, number of days when physical health was not good in the past 30 days, number of days when mental health was not good in the past 30 days, and number of days in which their usual activity was limited because of either poor physical or mental health in the past 30 days. As in prior studies, total number of unhealthy days was calculated as the sum of number of days when either physical or mental health was not good (maximum of 30 days). This HRQoL-4 tool has been shown to have strong psychometric properties and has been validated among many patient populations [20, 21].

### Other variables

Age, gender, race (non-Hispanic white, non-Hispanic black, Mexican-American, and other), poverty income ratio (PIR, as calculated by dividing family income by the poverty thresholds defined by the Department of Health and Human Services’ poverty guidelines) and disability (using the NHANES Activities of Daily Living [ADL] scale) [22] were assessed during in-person interviews. Age and PIR were standardized (mean = 0, SD = 1) for this analysis. Components of the NHANES ADL scale were categorized into 4 disability scales for this analysis: activities of daily living (ADL), instrumental activities of daily living (IADL), lower extremity mobility (LEM) and social activities (SA). Each scale is a sum of the number of activities in that scale in which the participant has some or much difficulty or is unable to do (the ranges for ADL: 0–3, IADL: 0–3, LEM: 0–5, SA: 0–3 activities).

### Statistical analysis

SAS survey procedures were used to account for the complex multistage sampling design of NHANES. Weighted means and standard errors were calculated for continuous variables, and weighted percentages were calculated for categorical variables. Laboratory weights were used since the sample was restricted to those with a fasting glucose. As in previous studies, we examined the prevalence of ≥14 unhealthy days by ideal CV health score [12, 15, 23]. We used logistic regression models to examine the association between CVHS category and fair/poor perceived general health. Poisson models were used to examine the association between CVHS category and the counts of total unhealthy days, days physical health was not good, days mental health was not good, and impaired activity days. For all outcomes, model I was adjusted for gender, race, standardized age, standardized PIR, and survey year, and model II was adjusted for all variables in model I plus ADL, IADL, LEM, and SA scores. Effect modification by race, gender, age ≥65 years and year of NHANES cycle was tested by including interaction terms with CVHS in each model. All analyses were conducted in 2014 using SAS 9.3 (SAS Institute Inc., Cary, NC). *P*-values less than 0.05 were considered statistically significant.

## Results

A total of 7115 NHANES participants from 2001 through 2010 were included. Among individuals without existing heart disease 14 % had an ideal CVHS (11–14), 46 % had an intermediate CVHS (8–10) and 40 % had a poor CVHS (0–7 points). Individuals with an ideal CVHS tended to be younger, male, and wealthier with a higher PIR than individuals with intermediate or poor CVHS (Table 2). They were also more likely to be non-Hispanic White, Other, and non-Hispanic Blacks as compared to Mexican American.

**Table 2:** Demographics and unhealthy days by CV health score category, NHANES 2001–2010

	Poor CV Health Score (Score 0–7)	Intermediate CV Health Score (Score 8–10)	Ideal CV Health Score (Score 11–14)	*p*-value
	*N* = 2848	*N* = 3248	*N* = 1019	
Age (years), weighted mean (SE)	48.8 (0.36)	42.6 (0.37)	37.0 (0.58)	<0.001
Male, weighted %	55.7	51.5	58.9	<0.001
Race, weighted %				0.002
Non-Hispanic white	34.8	47.6	17.6	
Non-Hispanic black	40.7	45.5	13.8	
Mexican American	38.4	47.8	13.8	
Other	29.7	49.3	20.9	
Poverty Income Ratio, weighted mean (SE)	2.9 (0.05)	3.2 (0.04)	3.4 (0.07)	<0.001
Disability score, weighted mean (SE)				
Activities of daily living	0.15 (0.01)	0.07 (0.01)	0.03 (0.01)	<0.001
Instrumental activities of daily living	0.18 (0.01)	0.09 (0.01)	0.04 (0.01)	<0.001
Lower extremity mobility	0.66 (0.03)	0.28 (0.02)	0.10 (0.01)	<0.001
Social activities	0.19 (0.01)	0.09 (0.01)	0.04 (0.01)	<0.001
Would you say in general your health is…, weighted %				<0.001
Excellent	5.6	12.9	24.6	
Very good	27.9	40.1	44.4	
Good	45.7	36.7	26.2	
Fair	18.6	9.1	4.7	
Poor	2.3	1.2	0.1	
Unhealthy days during past month, weighted mean (SD)	7.4 (0.23)	5.8 (0.19)	4.5 (0.26)	<0.001
For how many days during the past 30 days was your physical health not good?, weighted mean (SD)	4.2 (0.19)	2.7 (0.14)	2.0 (0.18)	<0.001
For how many days during the past 30 days was your mental health not good?, weighted mean (SE)	4.3 (0.17)	3.5 (0.15)	2.7 (0.20)	<0.001
For how many days did poor physical or mental health keep you from doing usual activities?, weighted mean (SE)	1.9 (0.14)	1.2 (0.11)	0.9 (0.09)	<0.001

**Fig. 1:**
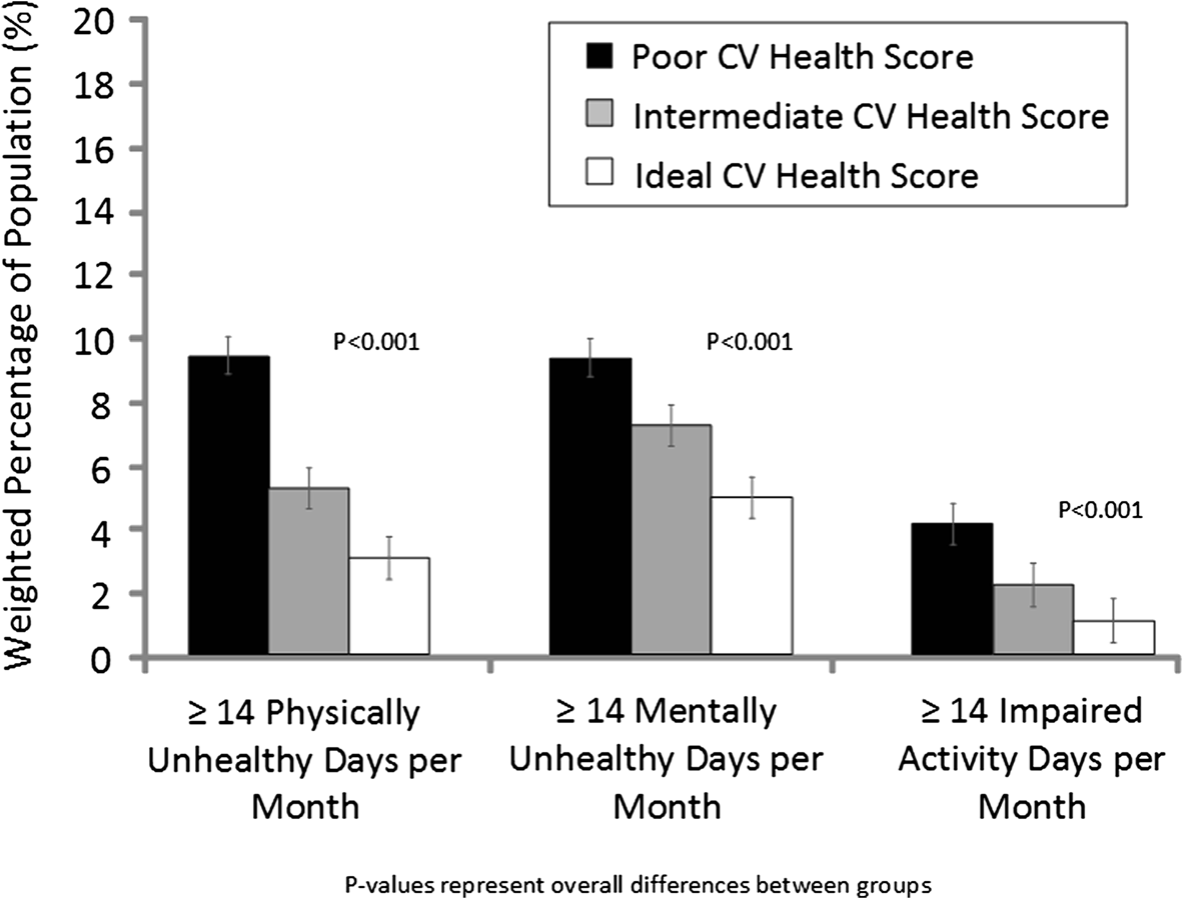
Prevalence of ≥ 14 unhealthy and impaired activity days by CVHS, NHANES 2001–2010. Note: *p*-values represent overall differences between groups

In unadjusted analyses, differences in overall health status were observed (*p*-value < 0.001), with the highest prevalence of good/very good/excellent health reported among those with high CVHS. The number of unhealthy days was also lower with greater CVHS (Table 2). Similar patterns were seen when physically unhealthy and mentally unhealthy days were examined separately, although mean differences between individuals in poor versus ideal CVH were larger for physical health than for mental health (mean difference was 2.2 days for physically unhealthy days and 1.6 days for mentally unhealthy days). In unadjusted analyses, individuals in poor CVH reported an average 1.9 days in which their usual daily activities were impaired because of their health in comparison to 1.2 days and 0.9 days among individuals with an intermediate and ideal CVHS respectively; *p*-value <0.001 for overall differences (Table 2). The proportion of individuals who experienced 14 or more physically or mentally unhealthy days was significantly lower for intermediate vs poor CV health and ideal vs intermediate CV health (Fig. 1).

**Fig. 2:**
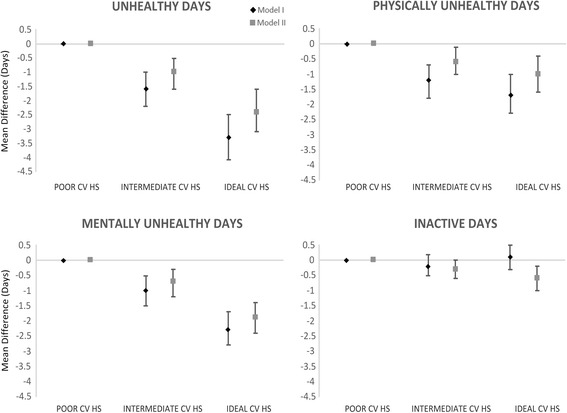
Adjusted^a^ mean difference and 95 % CI in unhealthy days^b^ by CVHS category, NHANES 2001–2010. ^a^Model I is adjusted for race, gender, standardized age, standardized PIR, and survey year; Model II is adjusted for all variables in Model I as well as Activities of Daily Living, Instrumental Activities of Daily Living, Lower Extremity Mobility, and Social Activities scores. ^b^per month

After adjusting for socio-demographics and disability (model II) significant differences remained in the number of physically unhealthy days, mentally unhealthy days, total unhealthy days, and the likelihood of being in fair/poor health (Fig. 2). As compared to those in poor CVH, individuals in intermediate CVH were 44 % less likely to report being in fair or poor health and individuals in ideal CVH were 71 % less likely to report being in fair or poor health. Similarly, individuals in ideal health reported 2.4 fewer unhealthy days in the past month as compared to individuals in poor CVH. Findings were consistent for both physically and mentally unhealthy days. For example, individuals in ideal CVH reported one day less of being physically unhealthy and almost two fewer days of being mentally unhealthy in the fully adjusted model (model II). The number of days in which either participants’ physical or mental health prevented them from performing their usual activities was lower for individuals in intermediate and ideal CV health adjusting for socio-demographics (model I). However, upon further adjustment for disability (model II), the association was attenuated.

**Fig. 3:**
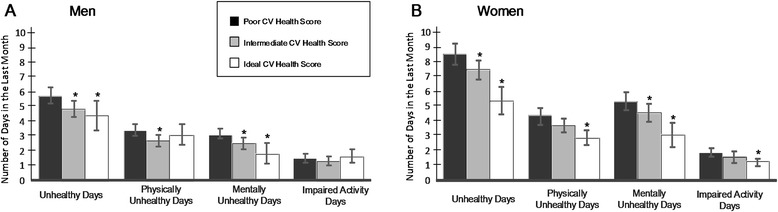
Adjusted^a^ mean unhealthy and impaired activity days^b^ by gender for each CVHS category, NHANES 2001–2010. ^a^Adjusted for race, gender, standardized age, standardized PIR, and survey year, Activities of Daily Living, Instrumental Activities of Daily Living, Lower Extremity Mobility, and Social Activities scores. ^b^in the last month. *p-value for comparison with mean days in poor CV health score category <0.05

The patterns described above were consistent by gender, race/ethnicity and age; however, significant interaction terms were identified for some outcomes. Adjusted mean differences in total unhealthy days and physically unhealthy days were more than 2 times larger for women in ideal CVH than for men in ideal CVH, 3.2 versus 1.3 fewer total unhealthy days than individuals with a poor CVHS in women and men, respectively (Fig. 3 and Additional file 1: Table S1). While women also had a greater number of mentally unhealthy days, the difference in overall unhealthy days between women and men was primarily driven by larger differences in physically unhealthy days among women compared with men (1.5 versus 0.3 fewer days, respectively). Significant interactions were also noted by age for the number physically unhealthy days and the number of days in which either physical or mental health kept participants from doing their usual activities (Additional file 1: Table S3). Younger individuals (<65 years of age) in ideal CVH reported 1.0 less physically unhealthy day in the last 30 days and 0.4 fewer days of impaired activity than individuals in poor CVH; in contrast, no significant differences were seen among older (≥65 years) individuals for either of these outcomes. After adjustment, no significant interactions by race/ethnicity or year (NHANES cycle) were identified (Additional file 1: Table S2).

## Discussion

Among a large, nationally representative sample, individuals in intermediate and ideal CVH reported better health status and HRQoL as defined by the number of physically and mentally unhealthy days within the past month compared to individuals with a poor CVHS. Individuals with an ideal CVHS reported 2.4 fewer unhealthy days over the past month as compared to individuals with a poor CVHS, and they were also 71 % less likely to report being in only fair or poor health. These findings were consistent for both physically and mentally unhealthy days, with women experiencing more physically and mentally unhealthy days than men.

HRQoL represents an important patient-centered outcome. Our results provide evidence that individuals in better CVH experience higher quality of life and fewer physically and mentally unhealthy days each month. These findings are consistent with studies of individual cardiovascular risk factors including smoking, diet, exercise, hypertension and metabolic syndrome with HRQoL [12–15]. This study provides new information on the association between overall CVH, namely ideal CVH as defined by the AHA, and HRQoL in the US population. Using NHANES data, our study extends previous research findings of an inverse association between self-reported CVD risk factor burden and HRQoL using both self-reported and directly measured physiologic data [24, 25]. Our findings suggest that ideal CVH is associated with HRQoL in multiple ways beyond simply decreasing the prevalence of CVD and disability. Further research is needed to explore the psychosocial mechanisms, such as optimism and resiliency, which may play a role in this association.

These findings are important not only at an individual level but also for the population. The indirect costs of CVD due to lost productivity is expected to grow dramatically over the next 20 years. By 2030, the projected total *annual* costs of CVD including the direct and indirect costs will exceed $1 trillion, including $275.8 billion in lost productivity costs [26]. Every day where poor physical or mental health keeps an individual from performing their usual activities translates to 0.312 missed work days with a loss of $341 (inflation adjusted) per missed work day [27]. Thus, our findings suggest that improving the CV health of workers might translate to reduced absenteeism and improved productivity for employers.

This study included a large, nationally representative sample of US adults with clinical and physical examination data. However, there are limitations to this study that should be considered. People with CVD were excluded from our analyses. However, people with CVD would be expected to have worse HRQoL than those without and therefore if they were included (in the poor CVHS), differences would be expected to be larger than observed. As with any cross-sectional study, we are limited in our ability to infer causality between the exposure and outcomes.

In conclusion, this study supports an association between ideal CVH and reduced number of physically and mentally unhealthy days, which may extend the benefits of improving CVH beyond reducing the incidence of CVD and disability. Primordial prevention, i.e. preventing the development of risk factors, could help achieve the goals of Healthy People 2020 and AHA’s 2020 Strategic Impact Goals by improving both the life expectancy and the quality of life for all Americans. These benefits are likely to extend beyond the individual to have a larger societal impact through reduced health care costs and lost productivity costs.

## Additional file

Additional file 1: Table S1. Association of Healthy Days and CV Health Score Category by Gender, NHANES 2001-2010. **Table S2.** Association of Healthy Days and CV Health Score Category by Race, NHANES 2001-2010. **Table S3.** Association of Healthy Days and CV Health Score Category by Age Category, NHANES 2001-2010. (DOCX 29 kb)

## References

[1] Go AS, Mozaffarian D, Roger VL, Benjamin EJ, Berry JD, Borden WB (2013). Heart disease and stroke statistics--2013 update: a report from the American Heart Association. Circulation.

[2] Nilsson PM, Nilsson JA, Berglund G (2006). Population-attributable risk of coronary heart disease risk factors during long-term follow-up: the Malmo Preventive Project. J Intern Med.

[3] Yusuf S, Hawken S, Ounpuu S, Dans T, Avezum A, Lanas F (2004). Effect of potentially modifiable risk factors associated with myocardial infarction in 52 countries (the INTERHEART study): case–control study. Lancet.

[4] O’Donnell MJ, Xavier D, Liu L, Zhang H, Chin SL, Rao-Melacini P (2010). Risk factors for ischaemic and intracerebral haemorrhagic stroke in 22 countries (the INTERSTROKE study): a case–control study. Lancet.

[5] Lloyd-Jones DM, Hong Y, Labarthe D, Mozaffarian D, Appel LJ, Van Horn L (2010). Defining and setting national goals for cardiovascular health promotion and disease reduction: the American Heart Association’s strategic Impact Goal through 2020 and beyond. Circulation.

[6] Folsom AR, Yatsuya H, Nettleton JA, Lutsey PL, Cushman M, Rosamond WD (2011). Community prevalence of ideal cardiovascular health, by the American Heart Association definition, and relationship with cardiovascular disease incidence. J Am Coll Cardiol.

[7] Ford ES, Greenlund KJ, Hong Y (2012). Ideal cardiovascular health and mortality from all causes and diseases of the circulatory system among adults in the United States. Circulation.

[8] Stamler J, Stamler R, Neaton JD, Wentworth D, Daviglus ML, Garside D (1999). Low risk-factor profile and long-term cardiovascular and noncardiovascular mortality and life expectancy: findings for 5 large cohorts of young adult and middle-aged men and women. JAMA.

[9] Daviglus ML, Liu K, Greenland P, Dyer AR, Garside DB, Manheim L (1998). Benefit of a favorable cardiovascular risk-factor profile in middle age with respect to Medicare costs. N Engl J Med.

[10] Rasmussen-Torvik LJ, Shay CM, Abramson JG, Friedrich CA, Nettleton JA, Prizment AE (2013). Ideal cardiovascular health is inversely associated with incident cancer: the atherosclerosis risk in communities study. Circulation.

[11] Reis JP, Loria CM, Launer LJ, Sidney S, Liu K, Jacobs DR (2013). Cardiovascular health through young adulthood and cognitive functioning in midlife. Ann Neurol.

[12] Bize R, Johnson JA, Plotnikoff RC (2007). Physical activity level and health-related quality of life in the general adult population: a systematic review. Prev Med.

[13] Ford ES, Li C (2008). Metabolic syndrome and health-related quality of life among U.S. adults. Ann Epidemiol.

[14] Froshaug DB, Dickinson LM, Fernald DH, Green LA (2009). Personal health behaviors are associated with physical and mental unhealthy days: a Prescription for Health (P4H) practice-based research networks study. J Am Board Fam Med.

[15] Hayes DK, Denny CH, Keenan NL, Croft JB, Greenlund KJ (2008). Health-related quality of life and hypertension status, awareness, treatment, and control: National Health and Nutrition Examination Survey, 2001–2004. J Hypertens.

[16] Daviglus ML, Liu K, Pirzada A, Yan LL, Garside DB, Feinglass J (2003). Favorable cardiovascular risk profile in middle age and health-related quality of life in older age. Arch Intern Med.

[17] DHHS DoHaHS. Healthy People 2020. http://www.healthypeople.gov/2020/topicsobjectives2020/overview.aspx?topicid=19.

[18] Huffman MD, Capewell S, Ning H, Shay CM, Ford ES, Lloyd-Jones DM (2012). Cardiovascular health behavior and health factor changes (1988–2008) and projections to 2020: results from the National Health and Nutrition Examination Surveys. Circulation.

[19] Centers for Disease Control and Prevention. Measuring Healthy Days. Atlanta, GA http://www.cdc.gov/hrqol/pdfs/mhd.pdf: November 2008.

[20] Dominick KL, Ahern FM, Gold CH, Heller DA (2002). Relationship of health-related quality of life to health care utilization and mortality among older adults. Aging Clin Exp Res.

[21] Dominick KL, Ahern FM, Gold CH, Heller DA (2004). Health-related quality of life among older adults with arthritis. Health Qual Life Outcomes.

[22] Cook CE, Richardson JK, Pietrobon R, Braga L, Silva HM, Turner D (2006). Validation of the NHANES ADL scale in a sample of patients with report of cervical pain: factor analysis, item response theory analysis, and line item validity. Disabil Rehabil.

[23] Brown DW, Balluz LS, Heath GW, Moriarty DG, Ford ES, Giles WH (2003). Associations between recommended levels of physical activity and health-related quality of life. Findings from the 2001 Behavioral Risk Factor Surveillance System (BRFSS) survey. Prev Med.

[24] Li C, Ford ES, Mokdad AH, Balluz LS, Brown DW, Giles WH (2008). Clustering of cardiovascular disease risk factors and health-related quality of life among US adults. Value Health.

[25] Jiang Y, Zack MM (2011). A latent class modeling approach to evaluate behavioral risk factors and health-related quality of life. Prev Chronic Dis.

[26] Heidenreich PA, Trogdon JG, Khavjou OA, Butler J, Dracup K, Ezekowitz MD (2011). Forecasting the future of cardiovascular disease in the United States: a policy statement from the American Heart Association. Circulation.

[27] Witter D, Agrawal S. Unhealthy U.S. Workers’ Absenteeism Costs $153 Billion 2011 September, 17, 2013.

